# MiRNA-494 enhances M1 macrophage polarization via Nrdp1 in ICH mice model

**DOI:** 10.1186/s12950-020-00247-3

**Published:** 2020-04-25

**Authors:** Gaohai Shao, Changlong Zhou, Kunlong Ma, Wang Zhao, Qijiang Xiong, Ling Yang, Zhongyan Huang, Zhao Yang

**Affiliations:** 1 Department of orthopedics, Yongchuan Hospital, Chongqing Medical University, Chongqing, 402160 China; 2 Department of Neurology and Chongqing key laboratory of cerebravascular disease, Yongchuan Hospital, Chongqing Medical University, Chongqing, 402160 China

**Keywords:** MiRNA-494, Macrophage, Polarization, Nrdp1, ICH

## Abstract

**Background:**

Ubiquitination-mediated M1/M2 macrophage polarization plays important roles in the pathogenesis of immune disease. However, the regulatory mechanism of ubiquitination during M1/M2 macrophage polarization following intracerebral hemorrhage (ICH) has not been well studied.

**Methods:**

In the experiment, macrophages were administered with erythrocyte lysates, and then miR-494-, Nrdp1-, and M1/M2-related markers were analyzed. Brain inflammatory response, brain edema, and neurological functions of ICH mice were also assessed.

**Results:**

We found that miR-494 levels increased while Nrdp1 levels decreased in macrophages after ICH. We also demonstrated that miR-494 inhibited Nrdp1 expression by directly binding its 3′-untranslated region. MiR-494 attenuated C/EBP-β activation and downstream proinflammatory factor production. Upregulation of Nrdp1 in macrophages significantly promoted M2 macrophage polarization via ubiquitinating and activating C/EBP-β. Moreover, the results indicated that miR-494 could enhance M1 macrophage polarization, promote brain edema, and impair neurological functions in ICH mice.

**Conclusions:**

Taken together, our results demonstrated that Nrdp1 contributed to M1/M2 macrophage polarization and neuroinflammation via ubiquitination and activation of C/EBP-β in ICH. miR-494 may provide a promising therapeutic clue for ICH.

## Introduction

Intracerebral hemorrhage (ICH) accounts for approximately 10 to 20% of all acute cerebrovascular diseases and is associated with high mortality and disability [1]. Preclinical and clinical evidence suggests that inflammatory response contributes to secondary brain damage following ICH, which is characterized by the accumulation and activation of inflammatory cells and production of inflammatory factors [2].

Much evidence suggests that macrophage-mediated inflammatory response plays an important role in hemorrhagic brain damage [3, 4]. Macrophages are highly plastic cells that can display diverse phenotypes and exert various functions according to specific microenvironmental signals [5]. M1 phenotype macrophages release proinflammatory mediators and leads to tissue damage. M2 phenotype macrophages generate anti-inflammatory cytokines and contribute to neuroprotective properties [6–8].

Ubiquitination is a common post-translational modification of protein and regulates many cell processes, such as the growth cycle and apoptosis [9–11]. Ubiquitination has also been identified in many aspects of immune responses [12–14]. Nrdp1, an E3 ubiquitin ligase, has previously been demonstrated to inhibit M1 activation of macrophages [15].

miRNAs are small non-coding (NC) RNA molecules (20-22 nucleotides) and contribute to mRNA post-transcriptional regulation [16–18]. In recent years, miRNAs have acted as potential biomarkers in inflammatory diseases [19–21]. In addition, miRNAs are involved in the development and regulation of both innate and adaptive immunity and contribute to the regulation of M1/M2 macrophage polarization [22–24].

However, the specific miRNAs regulating Nrdp1 and their potential role in M1/M2 macrophage polarization following ICH has not been studied. In the current study, we aimed to investigate whether miRNAs were involved in the regulation of M1/M2 macrophage polarization via Nrdp1 following ICH.

## Methods

### Animals

Eight-week-old male specific pathogen-free (SPF) C57BL/6 mice were purchased from Chongqing Medical University and were housed in standard polypropylene cages at the animal facility until the day of the experiment. The Chongqing Medical Experimental Animal Care Committee approved the protocol for this study, and all animal experiments were conducted in accordance with the National Institutes of Health Guidelines for the Care and Use of Laboratory Animals. All methods were carried out in accordance with relevant guidelines and regulations. All experimental protocols were approved by Chongqing Medical University. All University personnel who wish to use animals for teaching, research or experimentation must obtain ethical approval from Chongqing Medical University of Animal Ethics Committee prior to any use or involvement with animals. The animals were kept sedated during the course of the experiment. Mice were sacrificed by cervical dislocation at the end of the experiment. All animals received care in compliance with the Principles of Laboratory Animal Care and National standards.

### Macrophage culture

BMDMs (bone marrow-derived macrophages) were isolated from the marrow of the femurs and tibias of C57BL/6 mice. The legs of the animals were sprayed with 70% EtOH, and the skin and muscle tissue were removed from the bones. The bones were sprayed with 70% EtOH, transferred to a sterile-flow hood, and cut at both ends. The marrow was flushed out into a sterile falcon tube in Dulbecco’s modified Eagle’s medium supplemented with heat-inactivated fetal bovine serum (FBS; 50 ml; 10%) and penicillin-streptomycin (5 ml; 1%; Gibco). The cell suspension was triturated using a sterile Pasteur pipette, filtered through a nylon mesh filter into a sterile tube, and centrifuged (400×g, 5 min). The supernatant was removed, and the pellet was resuspended in red blood cell lysis buffer (Sigma-Aldrich, Gillingham). The suspension was centrifuged (400×g, 5 min), the supernatant was discarded, and the cells were washed using DMEM and centrifuged once more (400×g, 5 min). The pellet was resuspended in 20 ml of DMEM supplemented with L929-conditioned media (20%). Cells were seeded in sterile cell culture flasks (T175 cm^2^ flasks). On day 2, non-adherent cells were removed from the flask, the media was replaced, and the remaining adherent cells were maintained in culture for a further 6 days. The purity of the macrophage was over 95%.

### Neuronal cultures

Cortical neuronal cultures were prepared from embryonic day 16-18 C57BL/6 mice. Briefly, the cerebral cortices and hippocampi of fetal mice were dissected, and the meninges were carefully removed. Cells (1 × 10^6^ cells/mL) were maintained in poly-d-lysine (Sigma, St. Louis, MO)-coated plates in DMEM medium (Life Technologies) with 10% FBS (Life Technologies). After 4-6 h of culture, the cultures were replenished with neurobasal medium (Life Technologies) containing 100 U/mL of penicillin, 100 μg/mL of streptomycin, 2% B27, and 0.5 mM of glutamine (Life Technologies) at 37 °C with 5% CO_2_. The medium was changed every 3 days.

### Preparation of erythrocyte lysates

Whole blood collected from 30 to 50 mice was pooled and leukocytes were reduced using a Neonatal High-Efficiency Leukocyte Reduction Filter (Purecell Neo; Pall Corporation). Blood was centrifuged at 400×*g* for 15 min, and the volume was reduced to a final hemoglobin level of 17.0 g/dL, as determined by a modified Drabkin hemoglobin assay at a 1:251 dilution of stored red blood cells (RBCs) to Drabkin reagent (Ricca Chemical Company). Washed stored RBCs were prepared with 3 washes using 10 volumes of phosphate-buffered saline (PBS) and centrifugation at 400 *g*. After the final wash, the washed stored RBCs were resuspended in ddH_2_O to a final hemoglobin concentration of 17.0 g/dL. The white pellet of RBC ghosts was resuspended in PBS. Stroma-free RBC lysate was prepared by freeze-thaw of washed stored RBCs followed by centrifugation at 16,000×*g* to pellet and remove the stroma.

### Cell treatment

Macrophages (1 × 10^5^) were stimulated with 10 μl of PBS or erythrocyte lysates (a final hemoglobin concentration of 17.0 g/dL) for 48 h. After that, the supernatants were removed and further analyzed for cytokine production with enzyme-linked immunosorbent assay (ELISA).

### Real-time polymerase chain reaction (PCR)

The ipsilateral hemisphere was homogenized using RNAiso Plus (Takara) and ceramic beads for 1 min in a speedmill according to the manufacturer’s instructions (Alytik Jena). RNA was isolated according to the manufacturer’s instructions and reverse transcribed to obtain cDNA using a PrimeScript™ RT Reagent Kit with gDNA Eraser (Takara). Real-time PCR was performed using cDNA samples with SYBR@Premix ExTaq™II (Takara, Tli RNaseH Plus) by the One-step Plus analyzer (ABI). We normalized the results for each individual gene using the housekeeping gene beta-actin. The 2^−ΔΔCT^ method was used to calculate relative gene expression levels.

### Western blotting analysis

Proteins from cultured macrophages were resolved using sodium dodecyl sulfate polyacrylamide gel electrophoresis (SDS-PAGE) and transferred onto polyvinylidene fluoride membranes using electroblotting. The membranes were incubated with primary antibodies, all diluted to 1:1000 (Cell Signaling Technology), at 4 °C overnight. Glyceraldehyde 3-phosphate dehydrogenase (GAPDH, 1:200; Santa Cruz Biotechnology, Dallas, TX) was used as the loading control. The membranes were incubated with horseradish peroxidase (HRP)-conjugated goat anti-rabbit secondary Abs (1:2500; Sigma-Aldrich, St. Louis, MO) at 25 °C for 1 h. Bound Abs were visualized using a chemiluminescence detection system. Protein levels were calculated as the ratio of the target protein value to the GAPDH value.

### Elisa

The supernatants or brain tissue extracts were harvested, and tumor necrosis factor-α (TNF-α), interleukin (IL)-1β, and IL-6 productions were determined by ELISA. The specimens were assayed using respective ELISA kits (Minneapolis, MN, USA) according to the instruction manuals.

### Quantification of LDH in cell supernatants

The cytotoxic activity of macrophages was measured by a 6-h lactate dehydrogenase release assay using a CytoTox96 Non-radioactive Cytotoxicity Assay kit (Promega, Charbonnie’res-les-Bains, France) on 5 × 10^3^ neurons/well. Neurons were then added to the wells with 2 × 10^3^ macrophages. Experiments were performed in quadruplet, and the percentage of lysis was determined by OD490 measurement as described in the manufacturer’s instructions. Percentage cell lysis was calculated by the formula = [(Release of LDH from infected cells - Spontaneous LDH release) / (Maximum LDH release -Spontaneous LDH release)] × 100%.

Spontaneous release of LDH was obtained with untreated neurons, and maximum release of LDH was measured with lysed neurons by adding 1x lysis buffer supplied by the manufacturer.

### Oligonucleotide transfection

All of the transient transfections were performed with Lipofectamine 2000 Reagent (Invitrogen). MiRNA oligonucleotide transfections were performed according to an established protocol. Briefly, macrophages were seeded in 6-well plates at a density of 2 × 10^5^ cells per well and were grown overnight to 60-80% confluency. Next, miRNA mimic (Pre-miR™ miRNA precursor) or miRNA inhibitor (Anti-miR™ miRNA inhibitor) (Ambion) was added to the culture media at a final concentration of 100 nM according to the manufacturer’s recommendations. The following sequences of miR-494 were used: miR-494 mimic, 5′-UGAAACAUACACGGGAAACCUC-3′, miR-494 inhibitor, 5′-UUCUCCGAACGUGUCACGUUU-3′. Transfection efficiency (> 90%) was measured by quantitative reverse transcription PCR (qRT-PCR). Small interfering RNA (siRNA) inhibitors were purchased from Santa Cruz Biotechnology (Santa Cruz, CA) and were introduced into the macrophages at final concentrations of 100 nM according to the siRNA transfection protocol. Control siRNA-transfected macrophages were used as the negative control. Transfection efficiency (> 80%) was measured by qRT-PCR. After 6 h of transfection, the medium was replaced with normal glucose or low-serum (2% FBS) medium and cells were incubated for 48 h.

### Vector construction and luciferase reporter assays

Luciferase reporter constructs were used, and luciferase assays were performed as described previously. Briefly, the mouse Nrdp1 3′-UTR sequence was amplified by PCR from mouse genomic DNA and ligated into the pMIR-REPORT luciferase vector multiple cloning site (Ambion, Austin, TX) to yield pMIR-Nrdp1 3′-UTR (NRDP1 3′-UTR). Another pMIR-REPORT luciferase construct containing the Nrdp1 mRNA 3′-UTR with a mutation by site-directed mutagenesis was generated as a negative control and named Mut-Nrdp1 3′-UTR. Macrophages were plated in 6-well plates and allowed to reach 60-80% confluence overnight. Cells were then co-transfected with a reporter construct (pMIR-null REPORT plasmid, pMIR- Nrdp1 3′-UTR, pMIR-Nrdp1 3′-UTR-Mut). After 24 h, cells were harvested, and luciferase activity was measured using the Dual-Luciferase Reporter Assay System (Promega) according to the manufacturer’s recommendations. Luciferase activity was normalized to control TK Renilla construct expression (pRL-TK, Promega).

### Immunoprecipitation

Cells were lysed with radioimmunoprecipitation assay buffer (Cell Signaling Technology) supplemented with protease inhibitor mixture. Protein concentrations of the extracts were detected by BCA assay (Pierce). The lysates of NIH-3 T3 cells or macrophages were immunoprecipitated for 3 h with constant mixing at 4 °C with 2 μg/ml of anti-Nrdp1 or anti-C/EBP β antibody and protein A-agarose beads (Sigma) as indicated. After extensive washing with lysis buffer, the immunocomplexes were boiled in 2× loading buffer (Sangon, Shanghai) and subjected to SDS-PAGE, followed by immunoblotting.

### Assay of luciferase reporter gene expression

The mixture of Arg1 luciferase reporter plasmid, pRL-TK-Renilla-luciferase plasmid, and the other indicated plasmids were co-transduced into NIH-3 T3 cells. After 24 h, the cells were administered with 10 ng/ml of IL-4. The Dual-Luciferase Reporter assay system (Promega) was utilized to analyze the luciferase activity. By dividing Firefly luciferase activity with that of Renilla luciferase, the data were normalized for transfection efficiency.

### ICH model

After anesthetizing mice with 1-3% isoflurane inhalation and ventilating them with oxygen-enriched air (20%:80%), we injected a total of 0.5 μL containing 0. 075 units of collagenase VII-S (No. C9572, Sigma, St. Louis, MO) at 0.1 μL/min into the left basal ganglion at the following coordinates relative to bregma: 0.8 mm anterior, 2 mm lateral, and 2.8 mm deep. The craniotomy was sealed with bone wax, and the scalp was closed with 4-0 silk sutures. Rectal temperature was maintained at 37.0 ± 0.5 °C throughout the experimental and recovery periods (DC Temperature Controller 40-90-8D; FHC Inc., ME). Sham-operated mice received the same treatment, including needle insertion, but collagenase was not injected.

### Intracerebroventricular injection

To investigate the effects of miR-494, miR-494 inhibitor, or miR-494 mimics (2 μg/2 μl) was pretreated with a single intracerebroventricular (i.c.v.) injection in the ipsilateral ventricle 15 min before ICH. For the injection into the ipsilateral ventricle, a small burr hole was made in the parietal region (1.0 mm posterior and 1.0 mm lateral to the bregma) under the guidance of the stereotaxic instrument (RWD Life Science).

### Evaluation of neurological scores

A standardized battery of behavioral tests was used to quantify neurological function at 3ds post-ICH. The neurological scores were determined by Neurological Severity Scores. Neurological function was graded on a scale of 1-18. The higher the score, the more severe the injury (normal score 2-3; maximal deficit score 18). Tests were conducted by an observer blinded to the treatment group.

### Brain water content measurement

Brain water content was measured in mouse cerebral tissues after ICH. Briefly, mice were randomly sampled from each group and anesthetized by intraperitoneal injection with chloral hydrate (*n* = 5). Next, the cerebral tissues were removed, and the surface water on the cerebral tissues was blotted with filter paper. Brain samples were immediately weighed on an electric analytic balance to obtain the wet weight and then dried at 100 °C for 24 h to obtain the dry weight. Brain water content was calculated using the following formula: brain water content (%) = (wet weight - dry weight) / wet weight × 100%.

### Approvals

All experiments were performed following the relevant guidelines and regulations of the Chongqing Medical University. The methods were carried out in accordance with the approved guidelines. The study was approved by the ethics committee of Chongqing Medical University.

### Statistical analysis

All experiments were independently performed 3 times. The differences between groups were determined with the one-way analysis of variance (ANOVA) using SPSS 13.0 software. ANOVA test for independent measures is designed to compare the means of three or more independent samples simultaneously. *P* values of less than 0.05 were considered to be statistically significant.

## Results

### MiR-494 levels increased in erythrocyte lysate-treated macrophages and perihematoma tissue of ICH mice

**Fig. 1:**
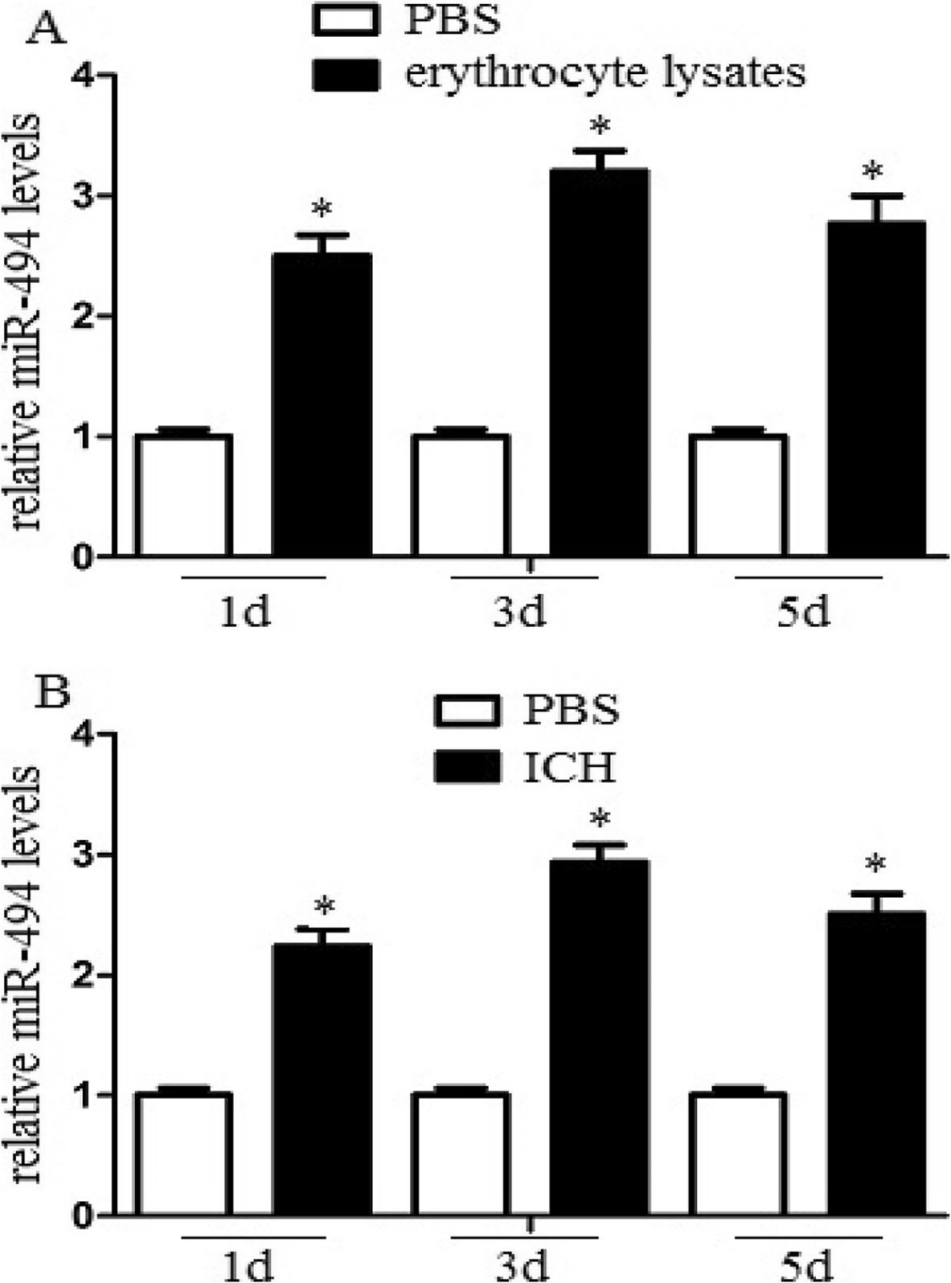
MiR-494 levels increased in erythrocyte lysates-treated macrophages and perihematomatissue of ICH mice. **a** Macrophages (1 × 10^5^) were administrated with 10 μl PBS or erythrocyte lysates for 1, 3, or 5 days. After then, the cell lysates were further analyzed for miR-494 mRNA levels. The level of miR-494 in erythrocyte lysates treated macrophages was much higher than that in PBS treated group at different time point in observation. *P < 0.05. **b** Mice were deeply anaesthetized and transcardially at 1, 3, or 5 days after ICH. The perihaematomal region of cerebral tissue was collected, and the tissue lysates were further analyzed for miR-494 mRNA levels. MiR-494 level in perihematoma tissue of ICH was much higher than that in sham group. Experiments performed in triplicate showed consistent results. The differences were analyzed using ANOVA. *P < 0.05

We used an erythrocyte lysate-treated macrophage model and experimental ICH model to detect miR-494 levels in vitro and in vivo. MiR-494 levels of macrophages or perihematoma tissue (The liquid accumulation surrounding ICH which appears as hypodensity around the hematoma on CT scan) were detected by real-time PCR following erythrocyte lysates treatment or ICH. We found that the level of miR-494 in erythrocyte lysate-treated macrophages was much higher than that in the PBS-treated group (Fig. 1a). In addition, we also found that miR-494 level in perihematoma tissue of ICH was much higher than that in the sham group (Fig. 1b). The data suggested that erythrocyte lysates and ICH promoted miR-494 levels.

### MiR-494 enhanced macrophage M1 polarization in vitro and in vivo

**Fig. 2:**
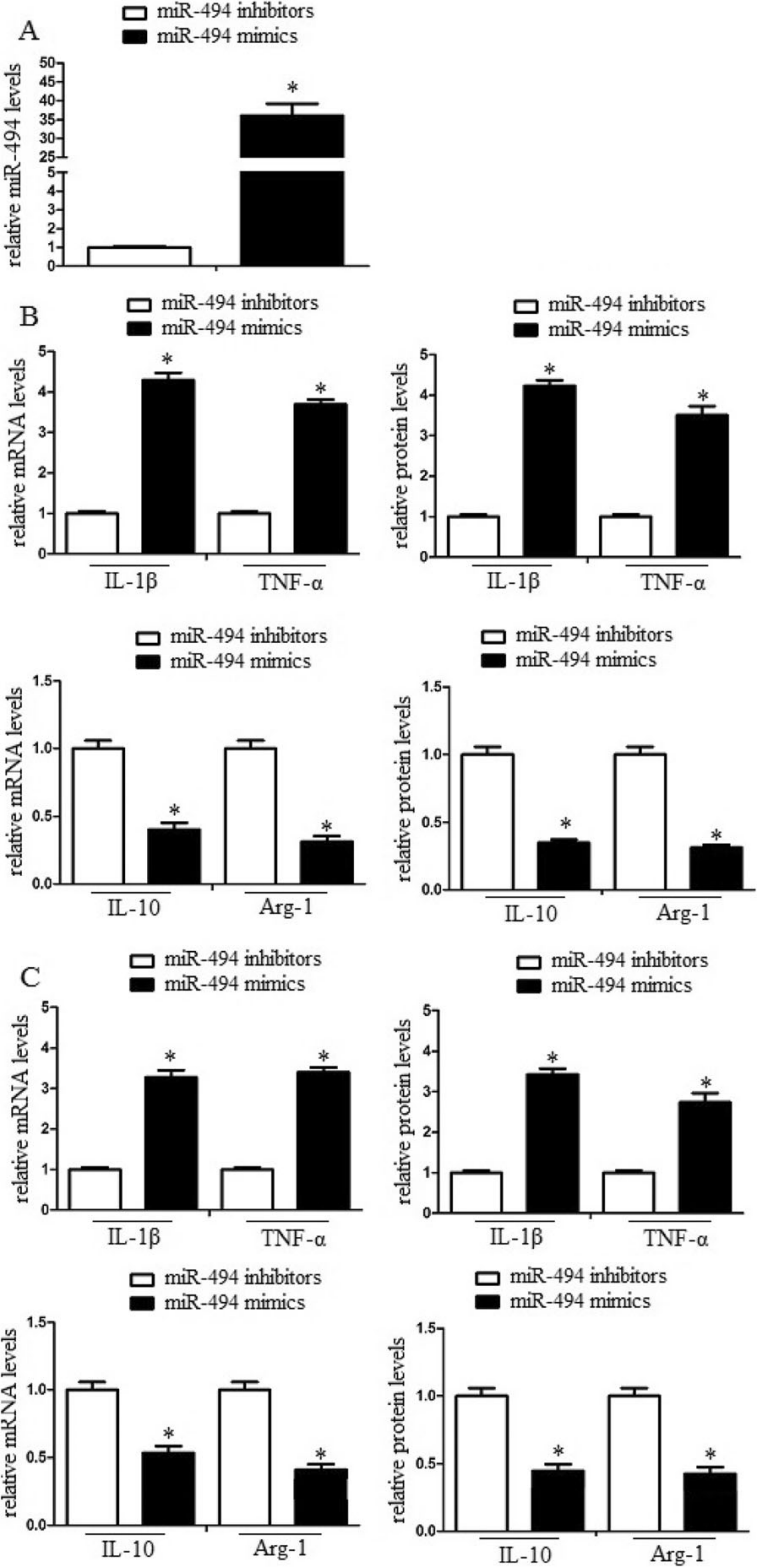
MiR-494 enhanced macrophage M1 polarization in vitro and in vivo. Macrophages were transduced with miR-494 mimics or inhibitors and then were treated with erythrocyte lysates for 3 days. **a** After then, the cell lysates were further analyzed for miR-494 mRNA levels by quantitative RT-PCR. **b** M1/M2 markers of the cell lysates were further analyzed by qRT-PCR and western blot assay. MiR-494 promoted M1 marker expression and inhibited M2 marker expression in vitro and in vivo. Experiments performed in triplicate showed consistent results. The differences were analyzed using ANOVA. *P < 0.05

To detect the effect of miR-494 on macrophage M1/M2 polarization in vitro, we detected M1/M2 markers in erythrocyte lysate-treated macrophages with qRT-PCR and western blot assays. Administration of miR-494 mimics or inhibitors was performed to upregulate or knock down miR-494 in macrophages. We found that miR-494 mimics significantly increased M1 marker IL-1β and TNF-γ levels compared with miR-494 inhibitors. In addition, miR-494 mimics significantly attenuated M2 marker IL-10 and Arg-1 levels compared with miR-494 inhibitors (Fig. 2b). The data demonstrated that miR-494 enhanced M1 marker expression, while it attenuated M2 marker expression in vitro. To further identify the effect of miR-494 on macrophage M1/M2 polarization in vivo, we detected M1/M2 markers in the perihematomal region of cerebral tissues. Intracerebroventricular injection of miR-494 mimics or inhibitors was performed to upregulate or knock down miR-494 in brain. We also identified that miR-494 promoted M1 marker expression and inhibited M2 marker expression in vivo (Fig. 2c).

### MiR-494 promoted inflammatory injury in vitro and in vivo

**Fig. 3:**
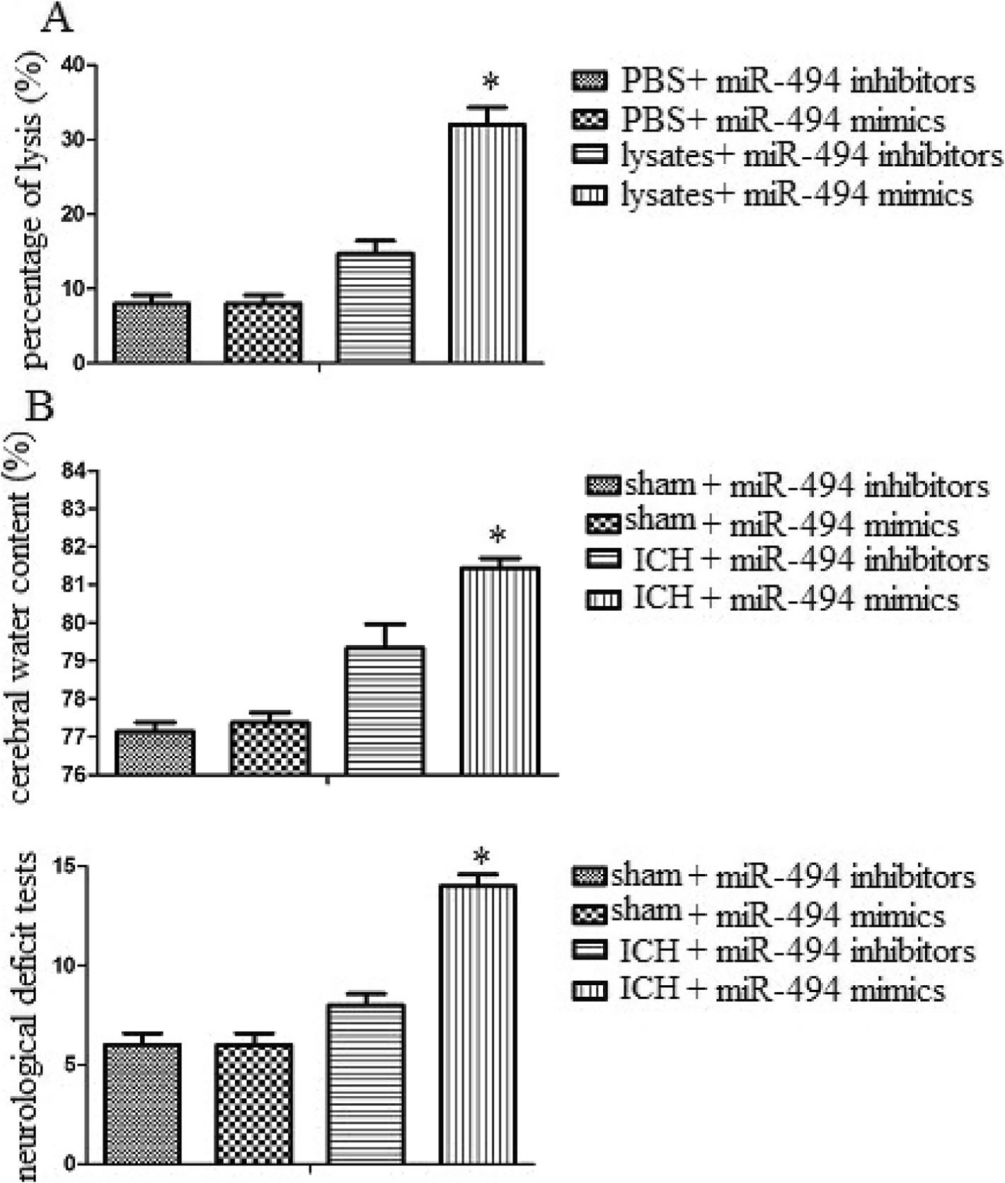
MiR-494 promoted inflammatory injury in vitro and in vivo. **a** Macrophages were transduced with miR-494 mimics or inhibitors, and then were treated with PBS or erythrocyte lysates for 3 days. The neurons were cocultured with a mixture of macrophage conditioned medium. Administration of miR-494 mimics significantly enhanced macrophage toxicity to neuron, while administration of miR-494 inhibitors significantly inhibited macrophage toxicity to neuron. **b-c** Intracerebroventricular injection of miR-494 mimics or inhibitors was administered 10 min after ICH. MiR-494 mimics significantly promoted water content and neurological damage, while miR-494 inhibitors significantly decreased water, content and neurological damage. Experiments performed in triplicate showed consistent results. The differences were analyzed using ANOVA. *P < 0.05

To explore the role of miR-494 on inflammatory injury, we utilized an LDH assay to detect the effect of miR-494 on macrophage toxicity to neurons. We found that administration of miR-494 mimics significantly enhanced macrophage toxicity to neurons. By contrast, administration of miR-494 inhibitors significantly inhibited macrophage toxicity to neurons (Fig. 3a). In addition, to explore the role of miR-494 to neurological function, i.c.v. administration of miR-494 mimics or inhibitors was performed 10 min after ICH. Brain water content and neurological injury of mice were observed 3 days after ICH. We found that miR-494 mimics significantly promoted water content and neurological damage. By contrast, miR-494 inhibitors significantly decreased water content and neurological damage (Fig. 3b). These data suggested that miR-494 could promote inflammatory injury in vitro and in vivo.

### Nrdp1 was a direct target of miR-494 in macrophages

**Fig. 4:**
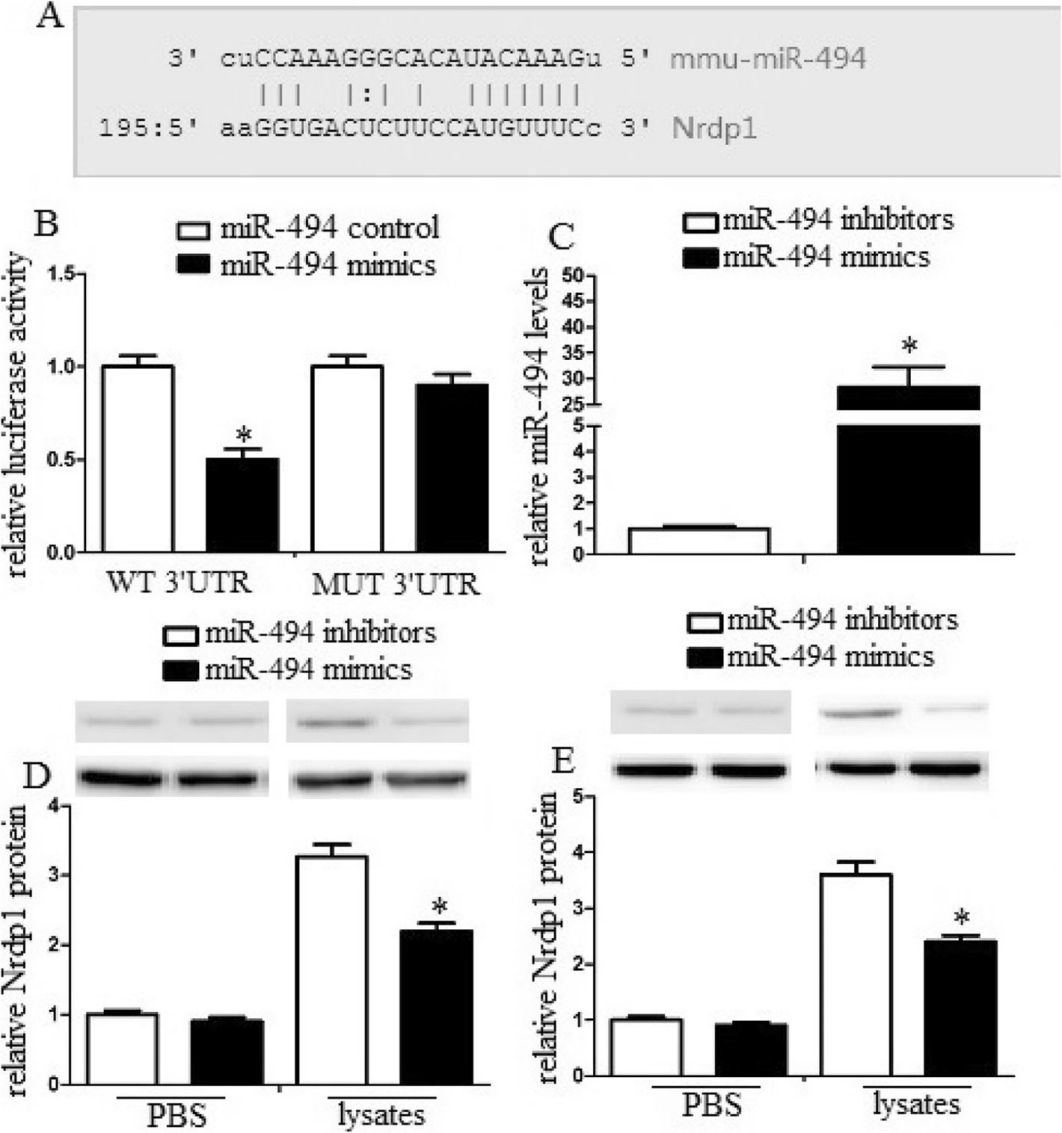
MiR-494 regulated Nrdp1˛levels in vitro and in vivo (**a**) Nrdp1 is a direct target of miR-494 in macrophage. The region of the Nrdp1 mRNA 3’UTR predicted to be targeted by miR-494 as indicated. **b** Luciferase activity assays using reporters with wild-type or mutant Nrdp1 3’UTRs were performed after cotransfection with miR-494 mimics or control in macrophage. **c** Macrophages were transfected with miR-494 mimics or inhibitors. After 24 h, cells were harvested, and miR-494 levels were evaluated by qRT-PCR. Transduction of miR-494 mimics enhanced miR-494 mRNA levels, while transduction of miR-494 inhibitors decreased miR-494 mRNA levels. **d** Macrophages were transfected with miR-494 mimics or inhibitors, and then were treated with PBS or erythrocyte lysates for 3 days. After then, neuron was treated with conditioned medium from treated macrophage. After 48 h, the Nrdp1 expression of macrophages was analyzed by western blot assays. **e** Intracerebroventricular injection of miR-494 mimics or inhibitors was administered 10 min after ICH. miR-494 mimics significantly attenuated Nrdp1 levels compared with control group. Experiments performed in triplicate showed consistent results. The differences were analyzed using ANOVA. *P < 0.05

The target prediction program TargetScan (www.targetscan.org) suggests 3′-UTR of Nrdp1 mRNA includes a putative miR-494 target sequence (Fig. 4a). To identify Nrdp1 as a direct target of miR-494 in macrophages, we analyzed this relationship by a dual-luciferase reporter system. Our data found that co-expression with miR-494 mimics significantly attenuated the activity of a firefly luciferase reporter containing wild-type Nrdp1 3′ -UTR, while miR-494 mimics could not attenuate the activity of a firefly luciferase reporter containing a mutated Nrdp1 3′-UTR (Fig. 4b). The phenomenon suggested that miR-494 likely attenuated Nrdp1 expression by directly binding target sites in the Nrdp1 3′-UTR.

### MiR-494 regulated Nrdp1 levels in vitro and in vivo

To explore the transduction efficiency, we transduced macrophages with miR-494 mimics or miR-494 inhibitors and detected miR-494 levels. The data demonstrated that transduction of miR-494 mimics enhanced miR-494 mRNA levels, while transduction of miR-494 inhibitors decreased miR-494 mRNA levels (Fig. 4c). In addition, to identify whether miR-494 could regulate Nrdp1 levels in vitro, we transduced macrophages with miR-494 mimics or miR-494 control and then treated macrophages with erythrocyte lysates to analyze Nrdp1 levels. The data suggested that miR-494 mimics significantly attenuated Nrdp1 levels compared with the control group (Fig. 4d). In addition, we analyzed Nrdp1 levels in the perihematomal region of cerebral tissues. The similar phenomenon of miR-494 was also detected in vivo (Fig. 4e).

### MiR-494 promoted macrophage M1 polarization and enhanced inflammatory damage via Nrdp1

**Fig. 5:**
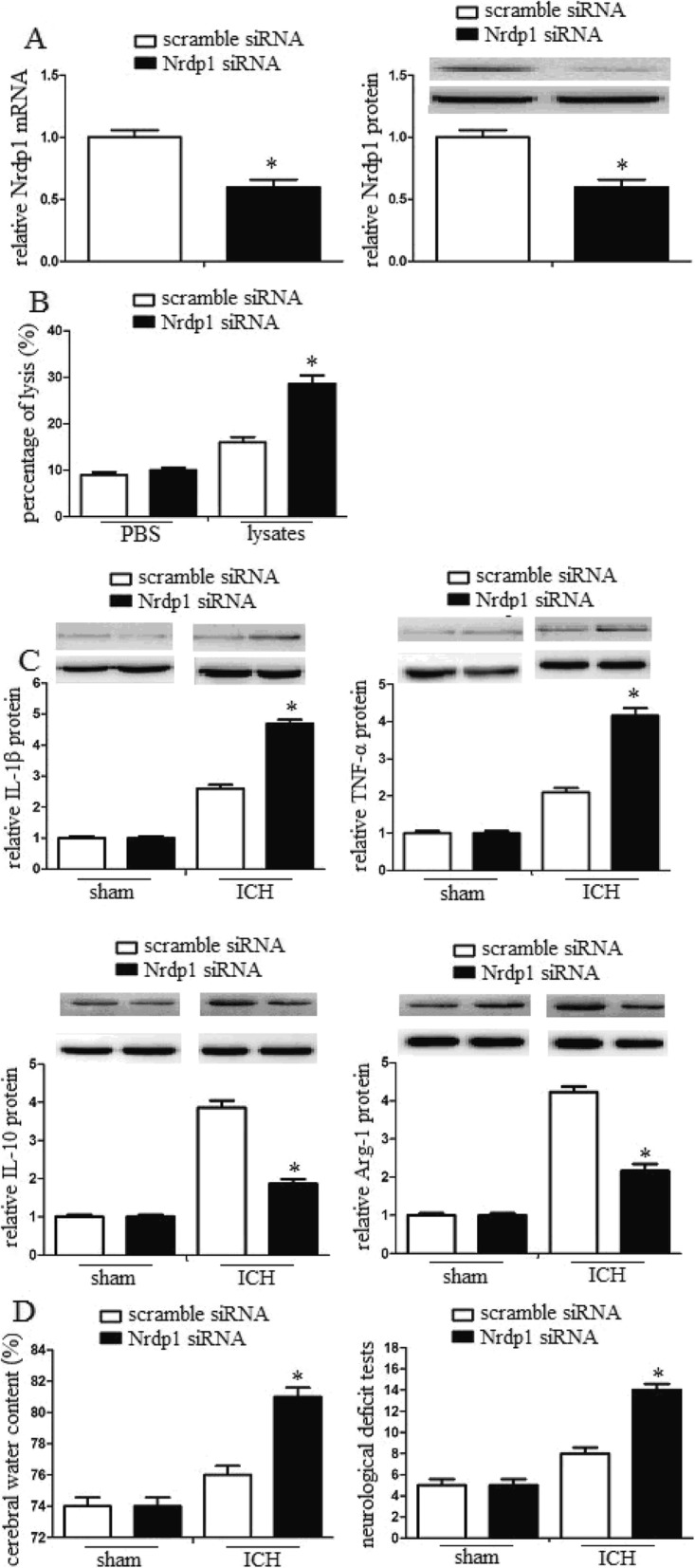
MiR-494 promoted macrophage M1 polarization and enhanced inflammatory damage via Nrdp1. **a** Detection of the inhibition efficiency of siRNAs against Nrdp1. Nrdp1 siRNA significantly attenuated Nrdp1 mRNA and protein levels. However, scramble siRNA did not attenuate Nrdp1 levels. **b** Macrophages were transfected with scramble siRNA or Nrdp1 siRNA, and then were treated with PBS or erythrocyte lysates for 3 days. After then, neurons were treated with conditioned medium from treated macrophage. Inhibition of Nrdp1 significantly promoted macrophage M1 polarization. **c** Intracerebroventricular injection of scramble siRNA or Nrdp1 siRNA was administered 10 min after ICH. Inhibition of Nrdp1 significantly attenuated neuron viability in vitro. **d** After 3 days of ICH, the cerebral water content of mice (*n* = 10) was also analyzed. In addition, the neurological deficit tests were performed by behavioral measurement. Inhibition of Nrdp1 significantly enhanced the cerebral water content and promoted neurological damage. Experiments performed in triplicate showed consistent results. The differences were analyzed using ANOVA. *P < 0.05

To assess the role of Nrdp1 in the miR-494 mediated inflammatory damage inhibition, we inhibited Nrdp1 levels and detected the inflammatory response of macrophages by siRNA assay. We found that Nrdp1 siRNA significantly attenuated Nrdp1 mRNA and protein levels. However, scrambling siRNA did not attenuate Nrdp1 levels (Fig. 5a). In addition, inhibition of Nrdp1 significantly promoted macrophage M1 polarization. However, scrambled siRNA had no such roles (Fig. 5b). Additionally, inhibition of Nrdp1 significantly attenuated neuron viability in vitro (Fig. 5c). Inhibition of Nrdp1 significantly enhanced the cerebral water content and promoted neurological damage (Fig. 5d). These data demonstrated that Nrdp1 contributed to miR-494 mediated inflammatory injury.

### Nrdp1 interacted with transcription factor C/EBP-β

**Fig. 6:**
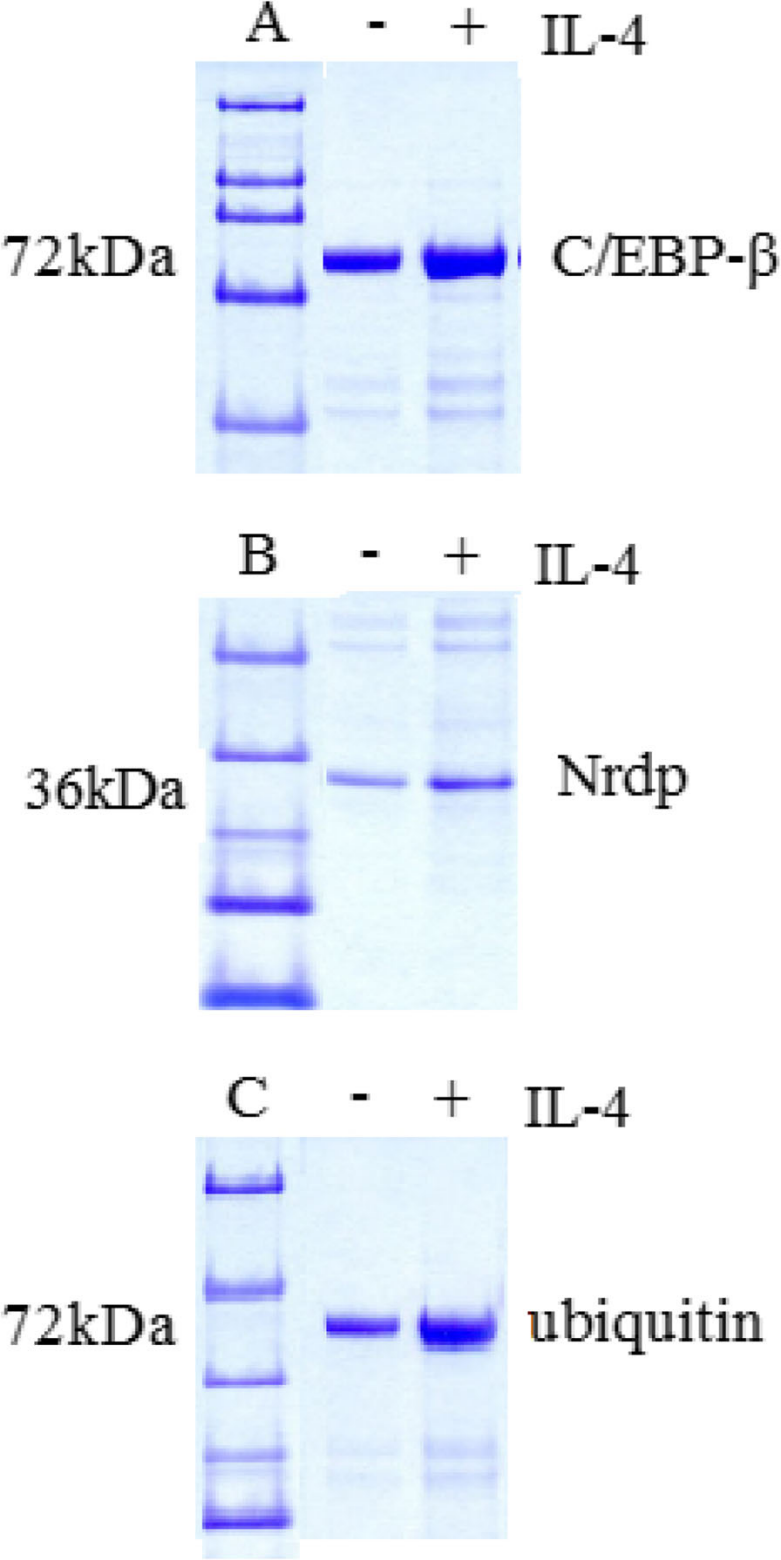
Nrdp1 interacted with transcription factor C/EBP-β. **a** NIH-3 T3 cells were transduced with pcDNA3.1-Nrdp1 and pcDNA3.1-C/EBP-β, and treated with or without IL-4 (10 ng/ml) for 1 h. Anti-Nrdp1 antibody could pull down C/EBP-β from NIH-3 T3 cell lysates and anti-C/EBP-β antibody could also pull down Nrdp1 from NIH-3 T3 cell lysates in the immunoprecipitant. **b** NIH-3 T3 cells were transduced with pcDNA3.1-Nrdp1 and pcDNA3.1-C/EBP-β, and treated with or without IL-4 (10 ng/ml) for 1 h. The interaction between Nrdp1 and C/EBP-β could be enhanced by IL-4 stimulation. **c** Macrophages were stimulated with 10 ng/ml IL-4 for 1 h. Cell lysates were immunoprecipitated with anti-C/EBP-β antibody. IL-4 stimulation induced the ubiquitination of C/EBP-β, and upregulation of Nrdp1 promoted ubiquitination of C/EBP-β

We also explored the regulation mechanism of Nrdp1 on macrophage M1/M2 polarization. Related evidence demonstrated that Arg1 was the key point to regulate macrophage M1/M2 polarization and C/EBP-β was essential transcription factor for IL-4-stimulated production of Arg1. Therefore, we identified whether Nrdp1 could enhance Arg1 gene transcription by regulating the activity of C/EBP-β. pcDNA3.1-Nrdp1 and pcDNA3.1-C/EBP-β were co-transduced into NIH-3 T3 cells, and immunoprecipitation assay was used to analyze the interactions between Nrdp1 and C/EBP-β. We found that anti-Nrdp1 antibody could pull down C/EBP-β from NIH-3 T3 cell lysates, and anti-C/EBP-β antibody could also pull down Nrdp1 from NIH-3 T3 cell lysates in the immunoprecipitant. Moreover, the interaction between Nrdp1 and C/EBP-β could be enhanced by IL-4 stimulation (Fig. 6a and b). These results suggested that Nrdp1 interacted with C/EBP-β.

### Nrdp1 promoted IL-4-induced ubiquitination of C/EBP-β

Nrdp1 is an E3 ligase that contributes to ubiquitination and also promotes ubiquitination in macrophages. To identify whether Nrdp1 regulated the ubiquitination of C/EBP-β, we detected the ubiquitination of C/EBP-β in macrophages. We found that IL-4 stimulation induced the ubiquitination of C/EBP-β, and upregulation of Nrdp1 promoted ubiquitination of C/EBP-β (Fig. 6c). Thus, E3 ubiquitin ligase Nrdp1 promoted the ubiquitination of C/EBP-β.

### Nrdp1 promoted transcriptional activity of C/EBP-β

**Fig. 7:**
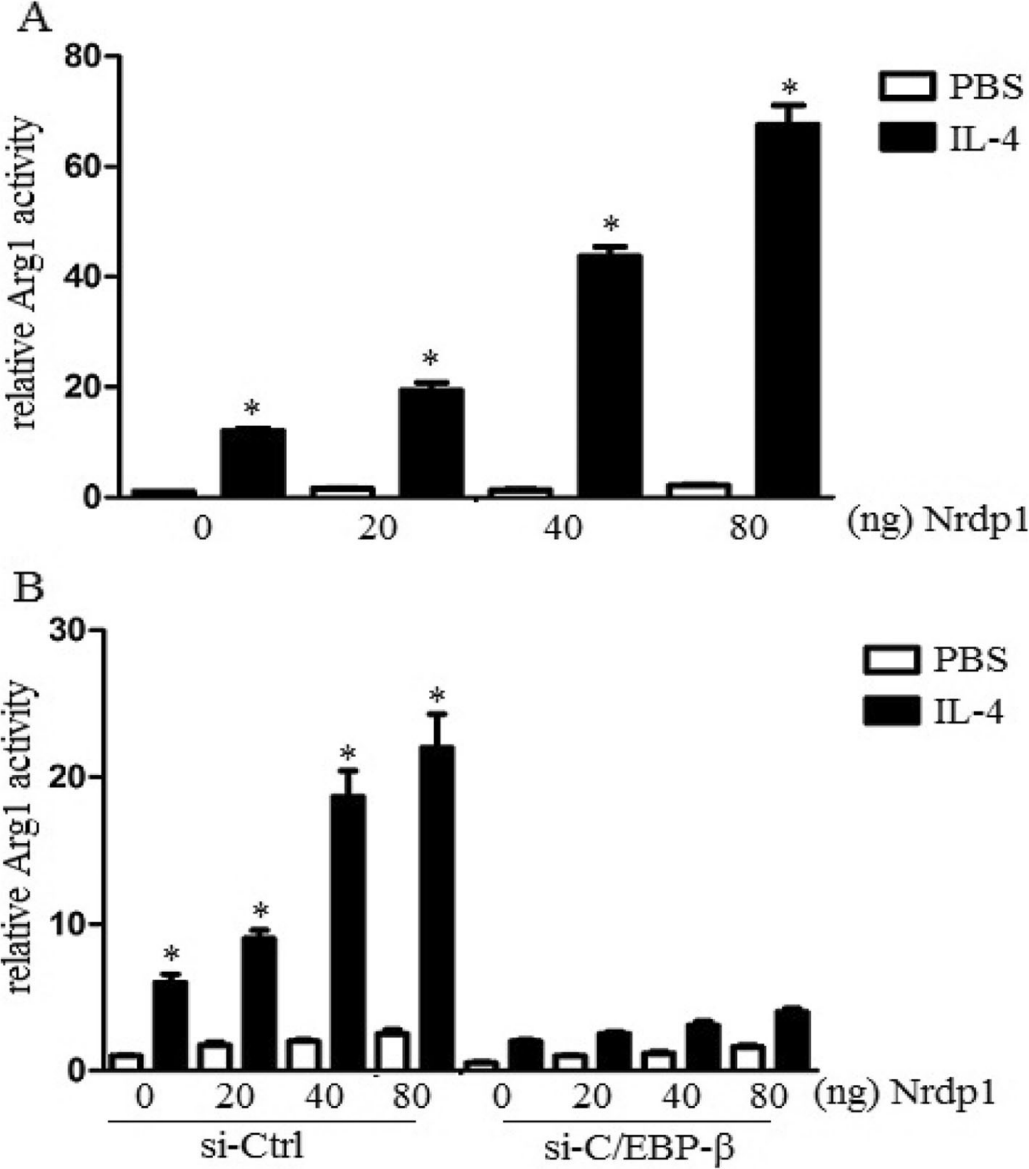
Nrdp1 promoted transcriptional activity of C/EBP-β. **a** Arg1 reporter activity in lysates was analyzed by Dual-Luciferase reporter assay system. Upregulation of Nrdp1 increased Arg1 reporter gene activation with a dose-dependent manner in the presence of IL-4 stimulation. **b** Arg1 reporter activity in lysates was detected, and luciferase activity was normalized to Renilla luciferase activity and was presented relative to basal luciferase activity. Knock down of C/EBP-β significantly attenuated Nrdp1-enhanced Arg1 gene activation. The differences were analyzed using ANOVA. *P < 0.05

To explore whether Nrdp1 could promote the transcriptional activity of C/EBP-β, we co-transduced Arg1 luciferase reporter plasmid, pRL-TK-Renilla-luciferase plasmid, pcDNA3.1-C/EBP-β plasmid, and indicated amounts of pcDNA3.1-Nrdp1 plasmid into NIH-3 T3 cells and analyzed the effect of Nrdp1 on C/EBP-β-induced transcriptional activation of the Arg1 reporter gene. The results demonstrated that upregulation of Nrdp1 increased Arg1 reporter gene activation with a dose-dependent manner in the presence of IL-4 stimulation (Fig. 7a). In addition, we utilized siRNA to knock down C/EBP-β expression in NIH-3 T3 cells and observed the role of C/EBP-β on Nrdp1-induced activation of Arg1 gene. The data demonstrated that knock down of C/EBP-β significantly attenuated Nrdp1-enhanced Arg1 gene activation (Fig. 7b). Therefore, the results suggested that Nrdp1 promoted IL-4-mediated activation of Arg1 gene by increasing transcriptional activity of C/EBP-β.

## Discussion

Macrophage M1/M2 polarization have been identified in various central nervous system diseases, such as traumatic brain injury, spinal cord injury, and ischemic stroke [25–27]. The activation of macrophages occurs in the hemorrhagic brain and leads to inflammatory damage following ICH [28–30]. However, the specific mechanisms underlying macrophage polarization following ICH have not been well studied.

Nrdp1 is a Ub ligase that promoted ubiquitination and proteasomal degradation of ErbB3, a member of the epidermal growth factor (EGF) receptor family [31]. Nrdp1 plays an important role in the regulation of cell growth or development, since it is differentially expressed in mouse fetal and adult hematopoietic stem cells and progenitors [32]. Nrdp1 inhibited the production of proinflammatory cytokines but increased interferon-beta production in Toll-like receptor-triggered macrophages [33]. Knockdown of Nrdp1 expression effectively inhibited IL-4-induced expression of M2-related genes in macrophages. Moreover, Nrdp1 inhibited LPS-induced production of inducible NOS and pro-inflammatory cytokines TNF-α, IL-1β, and IL-6 in macrophages [15]. The evidence suggested that Nrdp1 played a crucial role in macrophage polarization.

Macrophages are important components of the immune system, play a vital role in innate or adaptive immunity [34]. The function of macrophages can be divided into classical activation (M1) and alternative activation (M2) macrophages [35]. MiRNAs are small non-coding RNAs with the capability to regulate gene expression and cellular function [36]. Various studies have identified miRNA expression profiles in M1 and M2 polarized macrophages. More specifically, miR-9, miR-127, miR-155, and miR-125b have been reported to promote M1 polarization while miR-494, miR-223, miR-34a and let-7c can induce M2 polarization in macrophages [37]. Therefore, miRNAs that regulate macrophage polarization may have therapeutic potential in immune-related diseases. However, the specific miRNAs regulating Nrdp1 levels in M1/M2 macrophage polarization and the underlying molecular mechanism following ICH has not been studied. In this study, we used a well-established cell and mouse model to investigate the status of macrophage polarization following ICH. We also examined the role of Nrdp1 in ICH-induced inflammation and brain damage.

Firstly, we used an erythrocyte lysate-treated macrophage model and experimental ICH model to detect miR-494 levels in vitro and in vivo and found that erythrocyte lysates and ICH promoted miR-494 levels. To detect the effect of miR-494 on macrophage M1/M2 polarization, we detected M1/M2 markers in erythrocyte lysate-treated macrophages and in the perihematomal region of cerebral tissues. We found that miR-494 promoted M1 marker expression while inhibited M2 marker expression in vitro and in vivo, and miR-494 could promote inflammatory injury in vitro and in vivo. Secondly, we predicted Nrdp1 was the target protein of miR-494 and identified that miR-494 regulated Nrdp1 levels in vitro and in vivo. Thirdly, to assess the role of Nrdp1 in the miR-494 mediated inflammatory damage inhibition, we inhibited Nrdp1 levels and detected the inflammatory response of macrophages by siRNA assay. We found that miR-494 promoted macrophage M1 polarization and enhanced inflammatory damage via Nrdp1. Lastly, we also explored the regulation mechanism of Nrdp1 on macrophage M1/M2 polarization. Immunoprecipitation assay was used to analyze the interactions between Nrdp1 and C/EBP-β. The results suggested that Nrdp1 interacted with C/EBP-β. Moreover, we found that Nrdp1 promoted ubiquitination of C/EBP-β and IL-4-mediated activation of the Arg1 gene by increasing C/EBP-β activity.

## Conclusions

Taken together, Nrdp1 contributed to M1/M2 macrophage polarization and neuroinflammation via ubiquitination and activation of C/EBP-β in ICH. miR-494 may provide a novel therapeutic strategy for ICH.

## Data Availability

Please contact author for data requests.
